# Comparison of single-nucleotide polymorphisms and microsatellites in detecting quantitative trait loci for alcoholism: The Collaborative Study on the Genetics of Alcoholism

**DOI:** 10.1186/1471-2156-6-S1-S5

**Published:** 2005-12-30

**Authors:** Helen Kim, Carolyn M Hutter, Stephanie A Monks, Karen L Edwards

**Affiliations:** 1 Department of Epidemiology, University of Washington, School of Public Health and Community Medicine, Seattle, WA, USA; 2 Department of Biostatistics, University of Washington, School of Public Health and Community Medicine, Seattle, WA, USA; 3 Department of Statistics, Oklahoma State University, Stillwater, OK, USA

## Abstract

**Background:**

The feasibility of effectively analyzing high-density single nucleotide polymorphism (SNP) maps in whole genome scans of complex traits is not known. The purpose of this study was to compare variance components linkage results using different density marker maps in data from the Collaborative Study on the Genetics of Alcoholism (COGA). Marker maps having an average spacing of 10 cM (microsatellite), 0.78 cM (SNP1), and 0.31 cM (SNP2) were used to identify quantitative trait loci (QTLs) affecting maximum number of alcoholic drinks consumed in a 24-hour period (lnmaxalc).

**Results:**

Heritability of lnmaxalc was estimated to be 15%. Multipoint variance components linkage analysis revealed similar linkage patterns among the three marker panels, with the SNP maps consistently yielding higher LOD scores. Robust LOD scores > 1.0 were observed on chromosomes 1 and 13 for all three marker maps. Additional LODs > 1.0 were observed on chromosome 4 with both SNP maps and on chromosomes 18 and 21 with the SNP2 map. Peak LOD scores for lnmaxalc were observed on chromosome 1, although none reached genome-wide statistical significance. Quantile-quantile plots revealed that the multipoint distribution of SNP results appeared to fit the asymptotic null distribution better than the twopoint results.

**Conclusion:**

In conclusion, variance-components linkage analysis using high-density SNP maps provided higher LOD scores compared with the standard microsatellite map, similar to studies using nonparametric linkage methods. Widespread application of SNP maps will depend on further improvements in the computational methods implemented in current software packages.

## Background

Alcoholism is a complex trait influenced by both genetic and nongenetic factors. Previous linkage studies have identified several chromosomal regions harboring potential genes for alcoholism [1], although only a few have been replicated. Maximizing information in linkage studies will be crucial for the detection and replication of linkage signals.

Traditionally, studies involving genome-wide linkage scans have used microsatellite markers spaced evenly across the genome at ~10-cM intervals. An alternative and increasingly popular strategy is to use a high-density map of single nucleotide polymorphisms (SNPs). SNPs have several advantages over their microsatellite counterparts. They are found far more abundantly in the genome and are easier to genotype. Howerver, because of the lower heterozygozity on a per marker basis, a larger number of SNPs is necessary to achieve an information content similar to that of microsatellites [2].

One way of increasing power for linkage studies is to use more closely spaced markers, which is now possible with the advent of high-throughput technology for large scale SNP genotyping. Simulation studies have indicated that SNPs can offer equal or superior power to detect linkage compared with low-density microsatellite maps [2]. Recently, several studies have demonstrated empirically that denser SNP maps can improve gene localization and increase power to detect signals for complex traits, particularly in regions characterized by poor coverage or information content [3-5]. However, all of these studies used nonparametric linkage methods, and it is not known how SNPs will perform using other approaches, such as variance components, which can be more powerful than relative pair-based approaches. Thus, the purpose of this study was to compare variance components linkage results in detecting quantitative trait loci (QTLs) for alcoholism using different density marker maps.

## Methods

Data for the Genetic Analysis Workshop (GAW14) was obtained from the Collaborative Study on the Genetics of Alcoholism (COGA) [1]. Families with three or more members diagnosed with alcohol dependence were recruited from six COGA sites. Data were available from 143 pedigrees, including 1,614 family members. A subset of alcoholism phenotypes and covariates were provided. We chose the self-reported variable, "maximum number of drinks consumed in a 24-hour period," as our alcoholism phenotype. This quantitative trait is correlated with diagnosis of alcoholism and was previously shown to be linked to chromosome 4 in sibling pairs [6]. The trait was natural log transformed (lnmaxalc) to reduce skewness (2.41 vs. – 0.35) and kurtosis (12.19 vs. 2.82). Genome data included a standard 10-cM scan with 315 microsatellite markers and two SNP maps of differing densities. The Illumina Linkage III panel, referred to hereafter as SNP1, contained 4,752 SNPs with an average marker spacing of 0.78 cM. The Affymetrix GeneChip Mapping 10 K array (SNP2) contained 11,560 SNPs, averaging 0.31 cM. A total of 1,332 subjects had both microsatellite and SNP data available.

Heritability estimates and evidence for linkage were obtained using the variance components approach implemented in SOLAR version 2.1.2 [7]. This method partitions the total phenotypic variance into variation due to a major QTL, polygenic background, and random error. Under the null hypothesis of no linkage, the QTL variance is fixed at zero and is tested against a polygenic model in which the same parameter is estimated from the data using maximum likelihood methods. Quantitative genetic analysis of lnmaxalc after adjustments for age, sex, and ethnicity (coded as white, black, other) yielded a residual kurtosis of 1.07. Non-normally distributed traits may lead to excess type I error and inflated LOD scores in the variance components model [8]. Therefore, robust LOD scores were calculated within SOLAR by applying a correction factor (0.86886) based on 11,000 simulations of a fully informative marker, unlinked to the trait [8]. Two-point and multipoint identity-by-descent (IBD) probabilities were calculated using SOLAR [7].

We examined the two-point and multipoint genome-wide LOD score distributions of each marker set against the asymptotic null distribution using quantile-quantile (Q-Q) plots. Under the null hypothesis of no linkage, the likelihood ratio test statistic that is given by (2 *ln *10) × LOD is expected to be distributed as a 1/2:1/2 mixture of a  variable and a point mass at 0 [7]. To calculate empirical cutpoints for the maximum two-point and multipoint LOD scores for each marker set, we simulated 200–500 replicates of the chromosome 1 data using gene-dropping methods implemented in MERLIN version 0.10.2 [9]. Marker data are simulated under the null hypothesis of no linkage or association to the observed phenotype, keeping the marker informativeness, spacing, missing data patterns, and pedigree structure the same. These randomly generated datasets were then imported back into SOLAR for variance components analysis.

## Results

**Figure 1:**
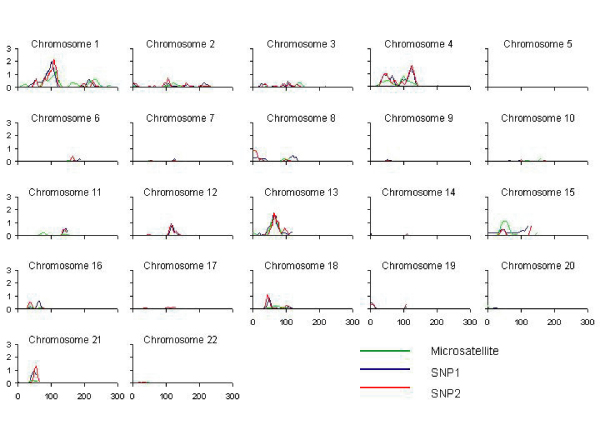
**Multipoint variance components LOD scores for log-transformed maximum number of alcoholic drinks per day (lnmaxalc), by marker panel. **Adjusted for age, sex, and ethnicity; cM = sex-averaged distance from p-term.

Heritability of lnmaxalc was estimated to be 15%. In general, genome-wide comparisons revealed similar linkage patterns among the three marker sets (Figure 1). The less dense SNP1 map appeared to perform similarly to the SNP2 map, although LOD scores were highest with the SNP2 map. Multipoint variance components linkage analysis revealed robust LOD scores > 1.0 on chromosomes 1 and 13 for all three marker sets (Table 1). Additional LODs > 1.0 were obtained on chromosome 4 with both SNP maps. Further signals on chromosomes 18 and 21 were observed with the densest SNP map (SNP2). Peak LOD scores for lnmaxalc were observed on chromosome 1 for all three maps.

**Figure 2:**
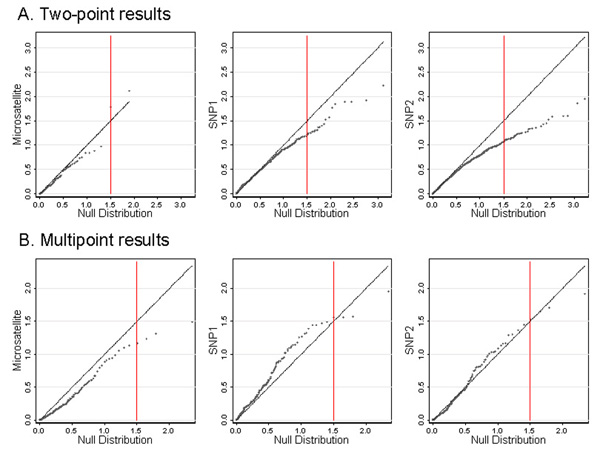
**Quantile-quantile plots of genome-wide LOD scores. **Twopoint (A) and multipoint (B) genomewide LOD scores are plotted against the asymptotic null distribution (1/2 ; 1/2 point mass 0) for each marker map. The red vertical line corresponds to a LOD score of 1.5.

Q-Q plots of genome-wide two-point and multipoint LOD scores are presented in Figure 2. The straight diagonal line represents the expected LOD score based on the asymptotic null distribution. A LOD score of 1.5 is indicated on each plot by a vertical red line. For the two-point results, the observed LOD scores were lower than expected (Figure 2A); this conservative bias was most pronounced for SNP2. In contrast, the SNP2 observed multipoint LOD scores, plotted every 5 cM, appear to fit the expected distribution better (Figure 2B).

To further explore the distribution of maximum LOD scores for each marker map, we simulated chromosome 1 data, where we obtained our highest genome-wide LOD scores, under the null hypothesis of no linkage. We generated 500 replicates for the microsatellite and SNP1 maps, and 200 replicates for the SNP2 map. The empirical LOD score cutpoints were consistently higher for the SNP maps than for the microsatellite map (Table 2). Our observed chromosome 1 LOD scores of 2.02 for SNP1 and 2.10 for SNP2 fall between the *P *= 0.05 and *P *= 0.01 cutpoints, whereas the microsatellite LOD of 1.26 corresponds to an empirical *P *> 0.10. A chromosome 1 *P*-value of 0.004 corresponds to a genomewide *P*-value of 0.05 . None of our LOD scores reached genome-wide level of significance.

## Discussion

A previous sib-pair linkage analysis of lnmaxalc in COGA found significant evidence for linkage (LOD = 3.5) on chromosome 4q21.3, near marker D4S2407 [6]. We were unable to replicate this finding in our dataset; however, we only had a subset of families and did not have the additional chromosome 4 microsatellite markers used in their study. Interestingly, our SNP-based analyses detected a weak signal in the same chromosome 4 region, suggesting that SNPs provided more information if indeed a true linkage signal exists. Instead, our peak LOD scores were found on chromosome 1, proximal to locations linked to other alcohol phenotypes [1].

Genome-wide two-point results from the three marker maps appeared to be comparable. However, the observed SNP-based LODs were lower than expected under the asymptotic null, which is probably due to the decreased information content of SNPs on an individual basis. For the multipoint results, the densest SNP map appeared to fit better, likely reflecting the increased information content provided by the larger number of markers or finer spacing. The asymptotic null distribution assumes independence across the genome, but LOD scores are correlated along a chromosome, even under the null hypothesis of no linkage. Our Q-Q plot comparisons may be affected by correlation among LOD scores. However, we would expect such correlation to cause greater deviations from the asymptotic null for the multipoint results rather than the two-point results as we observed.

Peak variance-components LOD scores were consistently higher for the SNP-based linkage analyses, similar to recent reports using nonparametric linkage methods [3-5]. However, we note that empirical *P*-values corresponding to a given LOD score may also be higher for the SNP-based analyses. To fully compare the power of marker panels, extensive simulations should be carried out under various genetic models, map densities and sample sizes.

Direct comparisons between SNP-based and microsatellite-based results in this analysis were hindered by several factors. First, we used the genetic maps as provided to us, which were not aligned among the three marker sets. Further, the presence of linkage disequilibrium among SNPs can lead to inflated LOD scores [5]. However, we did not test the hypothesis of both linkage and association, since the average marker spacing was 600 kb and 210 kb for the SNP1 and SNP2 maps, respectively. Another limitation is that we calculated multipoint IBDs using an approximation method. Accuracy of IBD estimation can influence the power to detect linkage [10]. We considered using Markov chain Monte Carlo-based and exact methods for IBD estimation but encountered difficulties when attempting to analyze the large number of markers in the SNP maps, i.e., programs either skipped larger families or performed computations too slowly. It was encouraging, however, to see that our multipoint results from the highest density SNP map fit the expected distribution well. Perhaps the increased information content compensated for the loss of information in the multipoint IBD approximation.

Moving to denser SNP maps, however, comes at the expense of increasing computational time. We performed analyses in SOLAR using the RAM drive rather than the hard drive, which decreased the computation time by ~50%. Each analysis of the simulated chromosome 1 data took approximately 23 minutes for the microsatellite map (38 markers), 3–5 hours for the SNP1 map (381 markers), and 15–19 hours for the SNP2 map (864 markers). At the time of our analysis, SOLAR was unable to handle more than 500 markers per chromosome, so we had to break the SNP2 data into two to three sections for the larger chromosomes. This complicated the analysis as we had to "bridge" across sections to minimize boundary effects when estimating multipoint IBDs. The latest release of SOLAR 2.1.4 can handle up to 2000 markers, but the computation time may still be quite substantial.

## Conclusion

In conclusion, variance-components linkage analysis using high-density SNP maps provided higher LOD scores compared with the standard microsatellite map. These results demonstrate that using dense SNP maps in linkage analysis is feasible and may increase power. However, the computational challenges are not trivial and will only increase as denser SNP sets become available. More widespread application of SNPs in linkage analysis will depend on further improvements to current statistical methods and associated software packages.

## Abbreviations

COGA: Collaborative Study on the Genetics of Alcoholism

GAW: Genetic Analysis Workshop

IBD: Identity by descent

Q-Q: Quantile-quantile

QTL: Quantitative trait locus

SNP: Single-nucleotide polymorphism

## Authors' contributions

All authors participated in the study design and read and approved the final manuscript. HK performed statistical analyses and drafted the manuscript. CMH performed statistical analyses and provided manuscript support. SAM and KLE guided the statistical analyses.

## Figures and Tables

**Table 1:** Regions of linkage to lnmaxalc with robust LOD scores > 1.0 in COGA families.^a^

Chromosome	Marker Panel	Marker Region	Map Position (cM)^b^	Multipoint robust LOD^c^
1	Microsatellite	D1S226	114	1.26
	SNP1	RS1492259-RS437749	101	2.02
	SNP2	RS0320943-RS1592272	108	2.10
				
4	SNP1	RS1495127-RS724950	121	1.44
	SNP2	RS0011540-RS0889153	124	1.66
				
13	Microsatellite	D13S800	64	1.52
	SNP1	RS768826-RS1023102	62	1.71
	SNP2	RS1391748-RS0045587	63	1.77
				
18	SNP2	RS0261494-RS0444635	43	1.09
				
21	SNP2	RS0422341-RS1653187	55	1.30

^a^Adjusted for age, sex, and ethnicity.

^b^cM, sex-averaged distance from p-term.

^c^Applied correction factor of 0.87 from 11,000 simulations.

**Table 2:** Chromosome 1 empirical maximum multipoint LOD score cutpoints.

	LOD score		
	
*P*-value^a^	Microsatellite^b^	SNP1^b^	SNP2^c^
0.1	1.29	1.53	1.44
0.05	1.48	1.85	1.69
0.01	1.98	2.46	2.25
0.005	2.07	2.72	2.54

^a^Chromosome 1 *P *= 0.004 corresponds to genome-wide *P *= 0.05.

^b^Based on 500 simulations.

^c^Based on 200 simulations.
